# Selections and Abstracts

**Published:** 1899-11

**Authors:** 


### SELECTIONS AND ABSTRACTS.

---

### THE ASSOCIATION OF HYSTERIA WITH ORGANIC DISEASE OF THE NERVOUS SYSTEM.*

The diagnosis of disease is the most difficult, and at the same time the most important, function of the physician. This is nowhere more apparent than in hysteria, a disease which may simulate almost any other, and where mistaken diagnosis may not only bring opprobrium upon the physician, but also do much mental and bodily harm to the patient.

The object of this paper is to call attention to the association of hysteria with organic disease of the nervous system, a not very

---

*Read at the meeting of the Mississippi Valley Medical Association, held in Chicago, October 3–6, 1899, by Philip Zenner, A.M., M.D., Cincinnati.

rare occurrence, which enhances the difficulties of diagnosis, not the less so that the fact of such association is too frequently not borne in mind by the practitioner.

We view hysteria now from a standpoint differing from that of former periods. The name is no longer a term of reproach. Hysteria is looked upon as a disease, not a disgrace. Nor is it diagnosed nearly so frequently as formerly, when the case was likely to be pronounced hysteria because it was not known what else to term it. To-day the diagnosis of hysteria is based upon the presence of well-recognized symptoms. A large part of what was termed hysteria has been found to be organic, or other well-recognized disease. The larger part is neurasthenia, a disease whose basis is nerve exhaustion. Hysteria, too, has as its basis, at least in many instances, nerve exhaustion, but the most important factor is the mental element. It is in large part a mental disease, its manifestations the direct result of suggestion or auto-suggestion.

A most interesting question in this relation is that of simulation of disease. There is no doubt that the word of the hysteric is not always reliable, and some conditions—for instance, alleged blindness of one eye, when it can be proven that the patient really sees with that eye—seem very perplexing. Yet it is generally conceded, to-day, that there is little conscious simulation in hysteria.

Let us dwell for a moment on the causes of hysteria, for it will help us to understand why it may occur with organic disease. The most important factor in etiology is heredity, though hysteria does occur without a neurotic history, and, apparently, even without the patients having formerly acquired a neurotic disposition. Among the not infrequent causes may be mentioned alcohol, syphilis, fevers and other debilitating conditions, trauma, shock, and the depressing emotions. It is well to note the same causes frequently produce organic disease of the nervous system. At the same time organic disease of the nervous system itself not infrequently causes hysteria. There are two factors that are especially effective here—fright or other painful emotional state that the actual disease evokes, and suggestion, the mental effect of present and anticipated symptoms. Let us add an instance of each.

Quite recently a young man came to me with locomotor ataxia. Only a few weeks before he had had the first intimation that he was not in perfect health, in the occurrence of a sudden diplopia. He learned from his physician that he was afflicted with a grave disease. The fright from this announcement brought on a marked hysterical mental state. It required strong assurances on my part that his condition was not as bad as he feared, and, finally, modified rest treatment, to entirely remove the hysterical manifestations.

Another case of locomotor ataxia, a man in middle life, who had had pronounced symptoms of that disease a number of years, was seized with attacks of astasia-abasia—inability to stand or walk for a few minutes. He continued to have such attacks for several months. They finally disappeared under the use of electricity, whose only influence, as applied in his case, could have been a suggestive one. The manner of the attacks and their cure, as well as the subsequent history, indicated that they must have been of an hysterical character.

Apart from the fact of a sometimes common etiology, and therefore coincidence of hysteria and organic disease of the nervous system, the latter may appear in old hysteria, for hysteria is no security against such a development. In such instances the physician is often caught napping, for not only are the hysterical manifestations likely to mask those of a graver disease, but the practitioner is usually not on the alert for the appearance of the latter. I will add a few illustrative cases.

A lady, fifty-three years of age, had hysterical manifestations for many years, during which time I saw her in consultation at varying intervals. At one time she believed herself paralyzed. At another time she had not written for months, and believed it was impossible that she could write until I gave her a firm order, when she did so in my presence, and seemed rejoiced to have that power. At another time she had the same apparent inability to read. She also had aphasic manifestations at different periods.

I have mentioned only a few of the many hysterical symptoms presented in course of years. Apparently the hysterical disease had been the result of fright and suggestion. Before the hysterical symptoms appeared she had some vertigo and headache, which her physician told her was due to a brain tumor, and that she had not long to live. Though future developments proved the incorrectness of this diagnosis, the idea was never altogether removed from her mind, and she was never again in a contented, easy state of mind, her normal condition.

This patient died of acute cerebral softening. The whole duration of her fatal malady was about six weeks. The symptoms were a very slow and steadily progressing paralysis, first in the arm, then in the leg, but never affecting the face; gradually increasing mental obtundity until toward the end there was profound coma; and the last few days of life a high temperature. There were several areas of softening, the chief one affecting the upper two-thirds of the central convolutions of one hemisphere.

At the very inception of the fatal malady the patient had a pain in the arm, and declared that the arm was paralyzed, notwithstanding that the strength of the arm at this time, and for several days thereafter, seemed to be normal. She was at this time also in a very hysterical frame of mind, laughing, crying, etc.

The diagnosis of her physician and of myself in the beginning was hysterical paralysis, a diagnosis that seemed well founded on account of the past history, the hysterical mental state, and the fact that a fixed idea of being paralyzed preceded the actual paralysis, as well as that the paralysis itself presented features to some extent characteristic of hysterical hemiplegia, gradual onset of the paralysis, and its absence in the face.

The correct diagnosis was finally forced upon us by the portentous progress of the case, but, notwithstanding that all the symptoms seemed to point so clearly to hysterical hemiplegia, there is no doubt that we would have made a correct diagnosis at an earlier period if the trend of our thoughts had not been influenced by the long anterior history of hysteria.

Quite recently I saw a patient where a similar history led a large number of physicians astray. She had had hysterical symptoms of various kinds for many years, but her condition had been decidedly worse six or eight months before I saw her, though no other diagnosis had been made. She came to me from a long distance, and, as is my routine custom, a thorough examination was made, and Bright's disease detected. The patient died some months later. During that time there was such a commingling of hysterical symptoms and those of uremia that it was often very difficult to determine the character of the manifestations presented.

The diagnosis in such instances may be a very perplexing problem. It is often sufficiently so in hysteria, which may simulate nearly every disease. As an instance I may state that I have seen meningitis taken for hysteria, and hysteria taken for meningitis, and that, too, by good men. Nor is the diagnosis any easier that the hysterical symptoms are likely to be manifested in a part where there is already actual disease—in a joint that has been injured or where there is a slight inflammation, in a larynx where there is a mild catarrh, and the like.

In the diagnosis we will be assisted by noting, firstly, the personality of the patient, age, sex, heredity, disposition, and history; secondly, the relations of symptoms to emotional conditions, for hysteria, or at least some of its manifestations, may appear at once from fright or other strong emotion, and subsequent symptoms may be influenced by like conditions; and thirdly, the clinical picture presented. The most characteristic features of hysteria are the hysterical temperament—in the lighter cases the chief condition presented—the stigmata of the disease—anesthesia, paralyses, contractures, limitation of the visual fields, hysterogenic zones, etc.—and the crises, which vary all the way from severe hystero-epileptic attacks to mere emotional storms. The diagnosis is sometimes helped by the peculiar grouping of symptoms, paralyses, etc., such as it would appear organic disease could not produce, or by rapid fluctuations in symptoms, their sudden appearance and disappearance, etc. Finally, the marked influence of mental impressions, the power of suggestion over the manifestations of hysteria, may throw a bright light on the nature of the disease.

There are some special symptoms that have a great diagnostic import—for instance, the *globus*, and concentric contraction of the fields of vision, for hysteria; and hemianopsia, alteration of the deep reflexes, rigid pupils, and the reaction of degeneration for organic nervous disease. Yet even these symptoms are not altogether pathognomonic, the first mentioned not at all, and hemianopsia, ankle clonus and rigid pupils have been found in hysteria.

The various diagnostic tests mentioned are not always found, nor are any of them invariably reliable. No wonder, therefore, that the most experienced and skillful diagnosticians have been led into error. The greatest safety lies in always bearing in mind that diagnosis is often difficult, that hysteria and organic disease may be present at the same time, and in a painstaking examination and careful observation of the case.

The importance of correct diagnosis is often very great. We would avoid the opprobrium of calling organic disease hysteria. On the other hand, we should not doom a case of hysteria by pronouncing it to be organic disease, a doom not only in name, but whose utterance may do untold harm to the patient. It is sometimes equally important that we should recognize the presence of each malady when both are present, especially because of the amenability of the functional disease to treatment. Not rarely, so far as the immediate condition of the patient is concerned, the functional trouble is the more important, and we have already seen that its appearance with organic disease is largely due to mental impressions, fright and suggestion. Hence the power of the physician in combating it by allayed fears, guarding against the ill effects of auto-suggestion incident to the organic disease, and inspiring the patient with new hope and courage. Here, as everywhere in the treatment of hysteria, awakening hope and cheer, and suggestive therapy, are the most powerful curative measures.—*Cincinnati Lancet-Clinic.*

---

### STOMACH TUBE.

In a paper on the above subject read by Dr. H. H. Roberts at the meeting of the Kentucky State Medical Society, and published in the *American Practitioner and News*, we find the following practical conclusions:

Let us speak briefly of the modern stomach-tube and the proper technique of its use. Tube which I prefer and believe to be the best on the market is a modification of Ewald's, which consists of a soft rubber tube about thirty inches long, to one end of which is attached a glass funnel; the other end is open and has two slits on either side half an inch long and one-fourth of an inch wide. Above the side openings are made a number of perforations going through the caliber of the tube and about one-sixteenth of an inch in diameter. The side slits prevent the tube from becoming occluded from mucus, and the small perforations act as a sprinkler, bringing the fluid into contact with a large portion of the stomach. The tube should have a white mark about sixteen inches from the stomach end, which is the average length from the teeth to the stomach.

There are many apparatus for washing the stomach, such as the Leube-Rosenthal apparatus, Hemmeter's double current stomach-tube, etc.; but as these apparatus are more adapted to the use of the specialist, we will not discuss their utility. The technique of using an ordinary stomach-tube is very simple. The first and most important procedure is to secure the utmost confidence of the patient; explain carefully the whole operation; do this even to the most minute details, and if the patient perchance should be unusually timid, an opportunity of observing with what ease a more experienced patient introduces the tube will be of valuable assistance. Avoid the use of all oils or greasy lubricants. Seat the patient in a straight-back chair, have him to lean his head slightly forward (most patients throw their heads backward), wet the tube with warm water, place back on the tongue, and tell the patient to swallow; the tube will readily enter the esophagus, when it can be rapidly passed into the stomach. Time may be saved with patients with tender fauces, especially those who smoke a great deal, by spraying the throat with a mild solution of cocain, 2 to 4 per cent., but this procedure will seldom be needed. After the tube has entered the stomach, avoid moving the tube, as this brings on nausea and vomiting. Have the patient breathe deeply and regularly, and attract his attention to something else by a pleasant conversation or some object. Plain warm water is the most desirable fluid to wash the stomach with. Many drugs have been recommended to use in the lavage, but I find that while many may be beneficial, it is best to use plain water for lavage, and afterward, if necessary to use drugs, to use Einhorn's gastric spray apparatus. By raising and lowering the funnel end of the tube the stomach can be filled and emptied many times without any discomfort to the patient.

The rapid strides which have been made in the knowledge of gastric diseases are due to the introduction of modern technique; we then, as physicians, should not abuse their valuable assistance nor let our patients abuse them. Under no circumstances should a patient be given a tube to use himself, and it should not be used by the physician until a positive diagnosis has been made by an examination of the filtrate of the stomach or other means. The stomach-tube should never be used in very weak patients or old persons while in the erect position, but treat this class of patients either in the recumbent or reclining position. The tube should never be used without a thorough examination of the patient's thorax. Penzoldt reports a case where the patient was to have had his stomach washed by means of the tube in the morning, but for some reason was delayed until the next morning; in the interval the patient died from the rupture of an aortic aneurism into the esophagus.

The use of the tube as far as gastric lavage is concerned is but seldom required. From a diagnostic standpoint the tube is indispensable. The indiscriminate washing of the stomach is capable of much harm, and is often positively injurious to the patient. In cases where there are large quantities of mucus present, the tube should be used, and a mild alkaline solution used in the fluid to facilitate the dissolving of the mucus. By requesting the patient to shake himself occasionally during the operation the fluid will be brought in contact with all parts of the stomach wall, and can be cleansed thoroughly. The tube should not be used more than every other day, or best two or three times a week. Use from one to three quarts of water or until the wash returns clean. In cases where there is stagnation of food in the stomach, resulting from either muscular weakness or stenosis, causing dilatation of the stomach, the use of the tube and lavage is of great benefit. Quite large quantities of fluid are often necessary to thoroughly cleanse these cases where there is marked dilatation. The tube is contraindicated in all constitutional cases, viz.:

1. Heart disease, especially where there is defective compensation.

2. Myocarditis.

3. Angina pectoris.

4. Fatty heart, especially the advanced stages.

5. Aneurism.

6. Recent hemorrhages, such as pulmonary, gastric, vesical, apoplexia, renal, rectal hemorrhage, etc.

7. Pulmonary tuberculosis.

8. Cerebral hyperemia.

9. Advanced cachexia.

10. In the continuous fevers, typhoid, remittent, etc.

11. Where there has been a recent hematemesis.

12. Ulcer of stomach.

13. Carcinoma of the pylorus, especially where there has been vomiting of the coffee grounds.

14. In all cases where there is a tendency for the gastric mucosa to bleed easily.

15. In those cases where the gastric affection is secondary to some more important primary disease.

The many conveniences, and especially the great aid the stomach-tube has given us in the exact methods of scientific analysis of gastric diseases, have, like all important therapeutic means, been abused, but will ever prove a benefit to suffering humanity in scientific hands, used in a scientific and judicious manner.—*Peoria Medical Journal.*

---

### PUERPERAL INSANITY—A CURSORY VIEW FOR THE GENERAL PRACTITIONER.

Dr. C. F. Macdonald (*Med. Rec.*, Feb. 18, 1899) says that the term puerperal insanity is somewhat loosely applied in text-books and standard authorities. Some recognize but a single type—puerperal mania, so-called—while others recognize three distinct forms of mental alienation, viz.: insanity of pregnancy, puerperal insanity in the narrow sense, and insanity of lactation. Both points of view, according to Macdonald, are unscientific and lead to inaccuracy.

The puerperal period begins with delivery and ends with the cessation of the lochia, and therefore covers a period of four to six weeks, and the notion of puerperal insanity should be restricted to that period.

Clouston's analysis of sixty cases showed that about 80 per cent. occurs within the first fortnight. Only one case occurred as late as the 28th day. From other reports of large material we find a consensus of opinion that the frequency diminishes in a ratio proportionate to the lapse of time post-partum.

Puerperal insanity should be divided into mania, melancholia, and dementia, the latter being rare except as a terminal stage of the other two. Puerperal insanity, then, is mania or melancholia accompanying the puerperal state.

Some authorities find that at least 90 per cent. of these insanity cases are examples of mania of relatively short duration, the remainder being melancholia, and essentially chronic and incurable; and Macdonald, after an independent investigation, agrees with this conclusion: Of twenty-nine case, twenty-two were examples of mania. Insane asylums show a smaller percentage of maniacal cases, which is natural, as many of these recover at home.

The causes of puerperal insanity are predisposing and exciting. Heredity is the most important of the former, and evidences of neuropathic or psycopathic taint will be found in a large majority of cases. Of other causes prolonged anxiety during pregnancy, such as is experienced by unmarried women, desertion by the husband, tedious labor, severe post-partum hemorrhage, etc., may be mentioned.

Of exciting causes, fright is most prominent. This may be excited by the sight of a still-birth child, by great loss of blood, etc. Some observers see evidences of an association with renal disease, albuminuria, toxemia, etc., and albumin is certainly always present in these cases. Combined etiological factors often occur in a case, while in some patients absolutely no causal moments can be found. All cases of insanity involve cerebral malnutrition.

Early recognition of puerperal insanity is of paramount importance, because it is possible that it may then be warded off, or mitigated, while the child may be removed from the possibility of injury at the hands of the mother. A change of disposition is suspicious, whether exaltation or depression be the result. Insomnia is almost invariably present, and, when associated with constipation and suppressed lochia just after delivery, should be regarded as a danger signal. There is also a peculiar change in the patient's expression.

Irritability, suspicion, aversion to the child develop, and finally delusions and hallucinations, chiefly of the visual and auditory type. Other mental troubles occur, but less constantly. Associated with these psychical changes are physical phenomena. The circulation of the extremities is very sluggish, and the latter are livid, puffy, cold and clammy. Both lochia and milk are often suppressed. There is constipation, scanty urine—which is often albuminous—a coated and tremulous tongue, great motor restlessness and suicidal and homicidal tendencies.

With regard to prognosis, fatalities are by no means rare; thus of the author's twenty-two cases of mania, four died, three of exhaustion, and the other of terminal dementia, while another one had not recovered when last seen. Seventeen of the cases had recovered.

Cases which exhibit acute delirium are likely to die of rapid exhaustion. A pulse somewhat above 100 is an unfavorable prognostic sign. The mania sometimes masks physical affections like peritonitis and cellulitis. In general, the prognosis as regards life is good. The prognosis of melancholia appears to be favorable on the whole.

The author believes that puerperal insanity may be aborted at times by hypnotics in free doses (sulphonal, trional, chloral). The vegetable varieties are much less used now than formerly, with the exception of opium and its preparations, which still remain the sheet anchor in melancholia. Deodorized tincture in doses of 15 to 20 minims three times daily is a favorite with the author in melancholia. In the latter form fluid extract cannabis indica and bromide of potassium are also useful. The exhaustion which accompanies nausea must be met by alimentation, forced if necessary, and by far the best form is fresh raw eggs combined with milk and cream. Good nursing and attention to the various functions complete the management.—*Obstetrics.*

---

### WOUNDS OF THE LIVER AND BILIARY TRACT.

The above was the title of a paper read by Dr. W. E. B. Davis at the recent meeting of the American Association of Obstetricians and Gynecologists. He said that penetrating wounds of the liver were not common. The surgeon may inflict such injuries in the treatment of hydatid cysts and abscesses of the liver. Severe wounds usually prove fatal from hemorrhage. There is often injury to the biliary canals of the liver and the extravasation of bile contributes to the fatal issue. He reported cases illustrating wounds produced by the surgeon.

One, a man aged sixty years, in which there was only half ounce of pus found in the right lobe of the liver, but in searching for this small cavity a wound of very considerable depth and great size, perhaps two inches in length, was inflicted. There was very profuse hemorrhage which was controlled by iodoform gauze packing. Great quantities of bile were discharged for three weeks. Patient made an excellent recovery, and he attributed this result not only to the drainage of pus, but to the opening up of some of the biliary canals which resulted in emptying of the liver.

Another case, a woman thirty years of age, in which there were attacks of pain, fever, and jaundice from a movable stone in the common duct; the liver became very much enlarged, and during an attack of peritonitis an incision was made, and the right lobe of the liver freely opened and packed with gauze. There was no pus in this case. Patient rapidly recovered. The liver returned to its proper size and the stone was passed some months after, when the patient was being prepared for a radical operation.

He also reported the case of a woman sixty years of age whose symptoms indicated obstruction of the common duct. The liver extended almost to the umbilicus. No nodules were to be made out on the surface. There being no obstruction in the common duct, it was decided that the obstruction was in the hepatic duct or its branches. A free incision was made into the right lobe of the liver with the hope of opening some of the biliary canals and thus relieving cholemia. Death occurred a few days after from exhaustion. The patient was almost in a dying condition at the time of the operation. A large malignant nodule was found at the autopsy in the transverse fissure which completely obstructed the branches of the hepatic duct.

He stated that wounds of the biliary tract beyond the liver produce death, not so much from the sudden escape of bile as from the continuous pouring of fresh bile into the peritoneal cavity. Gallons of fluid would not cause death if there were protective adhesions. He reported a case of gun-shot wound illustrating this point, where the patient was cured by repeated tappings—large quantities of bile being removed at the first operations.

He also reported experiments on animals where adhesions had given way after two weeks and death resulted from peritonitis.

He said that rupture of the liver might be limited to the upper or lower surfaces, or that the organ might be completely torn through, the parts being held together by the veins. In four cases in which he had operated on dogs and removed more than one-third of the liver, all died. In one case in which he removed the extreme left lobe of the liver the animal made complete recovery.

He reported six cases of operations on animals illustrating the fact that small quantities of bile could be injected into the peritoneal cavity without harm. In one case he injected as much as 5 drams of bile.

He reported twenty-three cases where he had operated on dogs to demonstrate the effect of bile in the peritoneal cavity. In those cases where there was only small escape of bile the animals recovered. Where there was considerable extravasation the animals died in from one to three days. The omentum and abdominal viscera in these cases would be highly bile stained, but there was not the redness of the intestines that is observed in septic peritonitis.

He claimed that the treatment of these cases consists in promptly opening the abdomen, controlling the bleeding by gauze packing and drainage. He reported a large number of cases in which he had produced wounds on the gall-bladder and ducts of dogs which had been successfully dealt with in this way. The opening in the bladder or duct will close as quickly as the fistula of a cholecystostomy.

He stated that it was not necessary to stitch the common duct after a removal of a stone. Gauze packing and a glass drainage-tube would protect the general cavity. He reported cases illustrating this.

---

### THE TREATMENT OF CONSTIPATION.*

Gentlemen :—During the present session of the school, which is now nearing its close, you have witnessed operations for nearly every known disease of the rectum. I am sure that you are convinced now, if never before, of the absolute necessity of giving some special study to this class of affections. I trust, too, that by this clinical demonstration you will have been profited sufficiently to do many of these operations, thereby relieving a large class of sufferers, a class, too, which has been wonderfully neglected in the past by the profession. You know how common it is for all such affections to be designated as "piles," and the patient to be assured that an ointment will effect a cure. Your experience here will prove to you what an error it is to so classify these troubles. You have seen at these clinics men and women whose lives have been wrecked by the want of proper treatment. Need I mention such formidable diseases of the rectum and colon as tuberculosis, syphilis and cancer, or the so-called minora ffections, as hemorrhoids, fistula, proctitis, ulceration, stricture, prolapse, polypoid growths, eczemas, pruritis, etc. Let me beseech you, therefore, not to look too lightly upon this class, but at least give them the benefit of a careful examination before you dismiss them. As the last clinic to be held this session I have summoned a number of patients who are not seriously ill, nor do they need any surgical operation. You see here some aged and some middle-aged, while here to my right is a very young person. Each one of these is a subject of that very common, and, what is generally regarded, very simple ailment—constipation. Before I begin to explain the condition of

*A clinical lecture delivered by Jos. M. Mathews, M.D., LL.D., Louisville, Ky., at the Hospital College of Medicine, Louisville, Ky.

these patients, or this class of patients, permit me to say that constipation is a relative term. What is constipation to one is not constipation to another. Very often you will hear a person say: "If my bowels do not move every day I feel badly—headache, languor and tired." Another, in apparent good health, will inform you that his or her bowels move only on every second, third or fourth day. The late Dr. D. W. Yandell once told me that a patient, in describing her trouble, said that so far as her bowels were concerned she was all right, as they moved with perfect regularity every two weeks. I have made mention to you of a case treated by me, and which is fully described in my work on "Diseases of the Rectum," a young lady whose bowels moved only once every three months, four times a year.

I do not wish you to be impressed with the idea, either, that constipation is a simple thing, for, to the contrary, it is often a very serious affair. I once heard an old physician say that "if his bowels moved in the morning he was sure that he would not die that day." As he is now dead, I have wondered "if his bowels moved that day."

Let us for a little time consider the physiology of defecation. The fecal mass has the cecum as its starting point, and when "a call of nature" takes place, it means that a peristaltic wave occurs, which moves this mass rapidly through the colon, dropping it into the sigmoid flexure, thence into the rectum. If the "call" is heeded by the individual an "action" is the result. If, through false modesty, attention to business or general laziness, attention is not paid to this effort of nature, then the watery constituent, which is the greater, is absorbed and carried into the circulation. In consequence we have an auto-infection which may prove of serious import. You can readily understand that by the absorption of the fecal mass, a poison, the whole general system would be deranged. The red corpuscles of the blood are diseased, altered in color and lessened in power. Hence a sallow complexion, dark rings under the eyes, cold extremities because of less supply of oxygen, lethargy due to vitiated blood and enfeebled corpuscles. The system is not nourished, hence the loss of flesh; the diseased blood circulates through the nervous system, and there is in consequence nervous depression—we might say nervous exhaustion—the pulse is slow and easily compressed; the organs of digestion and assimilation are lowered; there is loss of memory, no concentration of thought, and a great disposition to drowsiness. Notwithstanding that these patients are generally "sleepy," they are not relieved by sleep. All the functions are unsatisfactorily performed. If this condition is not relieved, disease and suffering must be the result. There is another phase of constipation that I would have you consider. We have stated that the liquid contents of the fecal mass is absorbed; the solid portion remains in the flexure and rectum. Daily and weekly this dried mass is added to, and in consequence we have the whole pelvic circulation deranged; external piles are produced, internal piles are made to bleed; atony of the coats of the bowel takes place; congestion, inflammation and ulceration may result. Truly, then, constipation is no "light" matter. What, then, shall we do for this condition? I once heard a doctor say that he would give a thousand dollars for a "specific" for constipation. I really believe the investment would have been a good one, when we consider how many people are thus affected.

Before attempting to map out any line of treatment, I wish to impress upon you that you should dignosticate between what is known as obstipation and constipation. The former may arise from a mechanical cause, as an irritable and contracted sphincter, a stricture or growth in the rectum, and some believe that the valves of the rectum play a part here. Of course, if either one of these conditions are detected you should turn your attention to their removal, for the obstipation is only secondary to them. I have relieved many cases of so-called constipation by dilating the sphincter muscle. But what should be done in a medical way to eradicate this condition? Let me say that you will find as most excellent adjuvants in the treatments of many of these patients: electricity, massage of the abdomen, cold baths and exercise. Every physician seems to have some favorite prescription, in the form of pill or solution, but they are constantly informed that "they have lost their power." Of course you have heard that the "regular habit" should be indulged in; that enemas are good under certain conditions, and a pill is necessary. But do such effect a cure? Very rarely. Each case must be studied as an individual one. Fat people as well as the lean are affected in this way—the young as well as the old. Women are more given to the habit than men, and I believe the reason to be that they are possessed of a womb. You will often find that a displaced uterus, or an enlarged one with adhesions, is responsible for the constipated condition. It is common with young schoolgirls, who, in the rush to get early to school, neglect the very important duty of having their bowels move in the early morning. Among the serviceable drugs in the treatment of this affection you will find the following: Cascara sagrada, sulphur, belladonna, nux vomicæ, sulph. iron, buckthorn, ipecac, magnesia, the mineral waters, and many others, either alone or in combination.

But let me impress upon you the necessity of making a more thorough study of such a case. If the patient who consults you is really desirous of getting well he should at least give you a fair chance to cure him. Supposing then that you have such consent, I would advise you to proceed in the following way: First try and ascertain what is the cause of the constipation. In this connection I wish to state that after an examination and observation of these cases extending over twenty years, I am forced to believe that a majority of them have as a basis constitutional derangement. In trying to solve the problem, it was observed that many of these patients were of a rheumatic or gouty diathesis. Acting upon this hypothesis, I have treated them by combating this special trouble and have found that in many cases the constipation would take care of itself. There are many preparations that you can use for this purpose, but the best is some form of lithia. Waters containing this salt will be found of service if taken in large quantities and for a long period of time. However, in my own practice I prefer to use the drug in a more concentrated form. I have, therefore, been using for some time a preparation of lithia known as thialion, with a marked degree of success. I direct that it be taken in teaspoonful doses, given in a full glass of hot water, taken before each meal. My theory is that in the rheumatic or gouty subject the intestines are brought under these same conditions that the disease or diseases are made manifest in other portions of the body. The muscular coat of the intestines is particularly affected by this gouty condition, and in consequence loses its contractile power. Anyway, I have cured patients of the confirmed constipation habit by this drug alone. To proceed, I would say to the patient that he must submit to my directions. You will find that in lieu of the rectal enema, that if a high enema is given through a Wales bougie, say of a half to a gallon of water, two or three times a week, it will be much more satisfactory. The object is to replace the amount of water which has been lost by absorption of the feces. A fruit diet, together with the drinking of large quantities of water should be enjoined. Massage of the abdomen by the patient himself, who should be taught the route of the colons, should be advised. The sweets should be forbidden and only plain, nutritious diet observed. I consider the administration of drastic purgatives harmful rather than beneficial. If you will watch this class of patients as carefully as you would any other chronic one, you will be rewarded by success. I beseech you not to get into the habit of prescribing for them in a routine way, for if you do they will soon desert you, and go elsewhere; besides, you will do them no good.—*Clinic.*

---

### SOME CAUSES OF RUPTURE OF THE UTERUS DURING PREGNANCY AND LABOR. 1. CAUSE WITHIN THE UTERUS.

Funck-Bretano (*Rev. prat. d' obst. et de pediatric*, Dec., 1898) first cites the statistics of Jolly (Thése de Paris, 1870) concerning the frequency of rupture of the uterus. Jolly found 230 cases of rupture in 782,741 confinements. The author does not regard these figures as trustworthy, and prefers to arrive at the frequency by taking the statistics of a single institution like the Clinique Baudelocque. From the date of inception of this clinic up to Dec. 1, 1898, out of a total of 17,662 confinements there were 8 cases of rupture, or 1 in every 2,207 labors, while in Jolly's miscellaneous statistics the proportion is 1 in every 3,403 labors.

Rupture of the uterus has become less frequent in proportion as the profession and laity better understand how to prevent some of the accidents of labor. Many cases of rupture are due to faulty positions, and nowadays even the women who are delivered at clinics present themselves before labor is due to see if the fetus is in a proper position.

Rupture is much more frequent during labor than before. Of 303 cases of rupture collected by Trask, 265 occurred at the time of delivery and 38 during gestation. Of these 38 cases only 12 occurred during the first six months of labor.

Rupture is divisible into traumatic and spontaneous, the latter being wholly independent of external violence, obstetric operations or the action of oxytocics.

During gestation the spontaneous rupture is much more common than the traumatic. In these spontaneous cases there is often an alteration in the uterine wall from fibroma, cicatrices or previous Cæsarean operation, invasion of the musculature of the uterus by a hydatiform mole, sphacelation of a portion of retroverted uterus, etc. In other cases of spontaneous rupture there is malformation (uterus bicornis).

Traumatic rupture occurs from external violence, such as a fall, compression of abdominal walls, penetrating wounds, instrumental procedures per vaginam, such as the use of Kiwisch's douche.

Traumatic rupture is more frequent in primiparæ (3 to 2), while in multiparæ the spontaneous form predominates. Multiparity is a predisposing cause, as rupture in primiparæ is infrequent (only 37 out of 455 cases). Other things being equal, the more children a woman has borne the more likely is she to have a rupture of the uterus. This is apparently due to a progressive weakening of both the uterus and abdominal walls which favors faulty positions, and especially an oblique direction of the uterus itself. Hecker's law that the fetus in each succeeding pregnancy tends to be larger and heavier than its predecessor also plays a part here.

Lacerations of the vaginal portion, so common in multiparæ, as well as cicatrices resulting from the same, also help to predispose this class of parturients to rupture.—*Obstetrics.*

---

### Mark Twain on Christian Science.

The horse doctor came, a pleasant man, and full of hope and professional interest in the case. In the matter of smell he was pretty aromatic, in fact quite horsey, and I tried to arrange with him for absent treatment, but it was not in his line, so out of delicacy I did not press it. He looked at my teeth and examined my hock, and said my age and general condition were favorable to energetic measures; therefore he would give me something to turn the stomach-ache into the botts and the cold in the head into the blind staggers; then he should be on his own beat, and should know what to do. He made me a bucket of bran mash, and said a dipperful of it every two hours, alternated with a drench of turpentine and axle-grease in it, would either knock my ailments out of me in twenty-four hours, or so interest me in some other ways as to make me forget they were on the premises. He administered my first dose himself, then took his leave, saying I was free to eat and drink anything I pleased, and in any quantity I liked. But I was not hungry any more, and I did not care for food.

I took up the Christian Scientist book and read half of it, then took a dipperful of drench, and read the other half. The resulting experiences were full of interest and adventure. All through the rumblings and grindings and quakings and effervescings accompanying the evolution of the ache into the botts and the cold into the blind staggers, I could note the generous struggle for mastery going on between the mash and the drench and the literature, and often I could tell which was ahead, and could easily distinguish the literature from the others when separate, though not when they were mixed, for when a bran mash and an eclectic drench are mixed together they look just like the Apodistical Principle out on a lark, and no one could tell it from that. The finish was reached at last, the evolutions were complete and a fine success, but I believe that this result could have been achieved with fewer materials. I believe the mash was necessary to the conversion of the stomach-ache into botts, and I think one could develop the blind staggers out of the literature itself; also, that blind staggers produced in this way would be of better quality and more lasting than any produced by the artificial process of a horse doctor.

For of all the strange and frantic, and incomprehensible, and uninterpretable books which the imagination of man has created, surely this one is the prize sample. It is written with a limitless confidence and complacency, and with a dash and stir and earnestness which often compel the effects of eloquence, even when the words do not seem to have any traceable meaning.—*Cosmopolitan.*

---

### TWELFTH CENSUS OF THE UNITED STATES. VITAL STATISTICS.

Physicians and students of mortality statistics will be interested in learning of the work now being accomplished by the Chief Statistician of Vital Statistics of the United States Census, by the authority of the Director, Hon. William R. Merriam. It is a practical effort, necessarily of limited scope, to secure the adoption of a uniform certificate for the return of deaths and looking toward the establishment of a common national system of collection of vital statistics for the purpose, primarily, of the census tables and publications.

Correspondence has been had by the Chief Statistician, Mr. William A. King, with the officers in charge of mortality registration in the States employing such a system, and in the cities having a population of 5,000 and more at the last census, which also collect and register death returns. Complete and accurate information of the different methods in vogue has been obtained, and it was found that there is much unnecessary and objectionable variation, considered from the census point of view, in the form of official returns.

Having no power to compel cooperative action, and hampered by want of time in which to carry out the whole project, nevertheless the Census Office undertook to secure the modification or amplification of the death certificates, so as to have them include the items necessary to obtain census data. A model return form was prepared and submitted, with explanatory correspondence, to each registration office or officer controlling the preparation of the State or local forms.

The result has been more important and gratifying than even the Census Office expected, as not only have the items in the specimen form been very generally adopted, but the registration officers have abolished many practically obsolete local variations in their certificates, and the latter have been made to conform to one standard more nearly than ever before.

The promptness and willingness displayed by the State and local officers in complying with the request of the Director has been surprising as well as gratifying. The benefit that will result to the Census Office and science from this first step toward the goal of national uniformity is incalculable, but it will be seen readily that the study of the natural law of the growth of the population is made easier and more certain.

The Director of the census confidently expects that physicians everywhere will appreciate the desirability of the new order of things, and that they will earnestly and actively cooperate in securing prompt and accurate mortality returns of the uniform character required by Congress and sought for by statisticians. He recognizes the fact that failure on the part of physicians to give vitality to the common standard by carefully reporting the items that may be new to their certificate will be fatal to the end in view.

---

### HOUSE TO HOUSE OPERATING.*

The skilled abdominal and gynecological surgeon of to-day is a product of surgical evolution, to which no man ever gave such an impetus as the great and lamented Mr. Lawson Tait. From him we have learned that the best results depend upon simplicity, thoroughness, rapidity, and rigid cleanliness; that his best work was done in house to house operating.

The advantages are many, and, briefly, are as follows: (1) The greater ability of the general practitioner to attend to the after-treatment, assisted by a competent nurse, the telegraph and telephone. (2) The absence of the mental dread of the patient to go into an institution, and the risk therein entailed, a condition by no means to be underestimated. (3) Iron bedsteads and improved household furniture are rapidly finding their way into country homes. (4) God's pure air, and undeniably, less liability to infection.

With reference to the use of antiseptics, it is unnecessary to mention but one fact, a quotation from Richelot, that "the elimination of all micro-organisms during the operation is to be condemned,

*Read before the American Association of Obstetricians and Gynecologists, Indianapolis, by Dr. Edwin Ricketts, Cincinnati, Ohio.

and in their stead we should rely upon hot water, a dry wound, and rapid operating, which means a shorter time under the anesthetic, a very important consideration.

---

### Beriberi in the United States.

Two cases of beriberi have been reported from Biloxi, Mississippi. These, with the recent case which was seen in Chicago, bring the case very much to the front. The nature of this disorder is not definitely understood, some writers classing it as a special form of endemic neuritis, due to malaria. Acute and chronic forms are described. A significant fact in the history of this disease is that it has never, so far as we know, appeared in this country, notwithstanding that malaria is quite as frequent in some parts of the United States as it is in the tropics. Malaria, neuritis and neuralgia are by no means infrequently associated in the United States; but there is a striking difference in the clinical appearance of these complications and cases of beriberi. Late observations lend strong support to the view that this disorder is due to a specific organism. Whether the disease is infectious or contagious has not been definitely determined, one writer attributing it to the eating of decayed rice, which would place it in the group of diseases to which palagra belongs. Intestinal parasites have been said to cause the disease. Ogata of Tokio says that it is due to a specific bacillus. The tissues chiefly affected are the peripheral nerves; but with our knowledge of malaria and its parasite, it is clear that it is not to be included among the malarial infections. It is certain that the disease will acquire some importance from the fact that cases will be distributed about the country by the returning troops from Manila.—*Medical Review.*

---

To prevent nausea and vomiting after ether anesthesia the inhalation of cider vinegar is recommended by many surgeons. Saturate a cloth or towel with strong vinegar and suspend it or hang it from the bedstead so that the patient will inhale the vapor constantly. It also relieves thirst and refreshes the patient.—*Journal of M. and S.*

